# Das RS3PE-Syndrom – eine seltene fakultative Paraneoplasie

**DOI:** 10.1007/s00393-025-01704-1

**Published:** 2025-08-27

**Authors:** Andriko Palmowski, Robert Biesen, Paula Hoff, Malte Lehmann, Rajan Somasundaram, Frank Buttgereit, Hildrun Haibel

**Affiliations:** 1 Medizinische Klinik mit Schwerpunkt Rheumatologie und klinische Immunologie, Charité – Universitätsmedizin Berlin, Charitéplatz 1, 10117 Berlin, Deutschland; 2 The Parker Institute, Section for Biostatistics and Evidence-based Research, Copenhagen University Hospital, Kopenhagen, Dänemark; 3 Berlin Institute of Health (BIH) at Charité – Universitätsmedizin Berlin, Berlin, Deutschland; 4 Epidemiologie und Versorgungsforschung, Deutsches Rheuma-Forschungszentrum (DRFZ), ein Leibniz-Institut, Berlin, Deutschland; 5 Endokrinologikum Berlin, Berlin, Deutschland; 6 Medizinische Klinik für Gastroenterologie, Infektiologie, Rheumatologie, Campus Benjamin Franklin, Charité – Universitätsmedizin Berlin, Berlin, Deutschland; 7 Zentrale Notaufnahme, Campus Benjamin Franklin, Charité – Universitätsmedizin Berlin, Berlin, Deutschland; 8 Translationale Rheumatologie, Deutsches Rheuma-Forschungszentrum (DRFZ), ein Leibniz-Institut, Berlin, Deutschland

**Keywords:** RS3PE, Rheumatoide Arthritis, Glukokortikoide, RS3PE, Rheumatoid arthritis, Glucocorticoids

## Abstract

Das RS3PE(„remitting seronegative symmetric synovitis with pitting edema“)-Syndrom ist eine seltene entzündlich rheumatische Erkrankung des älteren Menschen, die auch paraneoplastisch auftreten kann. Leitsymptom ist die rasch progrediente, symmetrische ödematöse Schwellung beider Hände, teils auch beider Füße, begleitet durch (Teno-)Synovitiden. Autoimmunserologien (Rheumafaktoren, Antikörper gegen zyklische citrullinierte Peptide, antinukleäre Antikörper) bleiben typischerweise negativ. Wird die Diagnose eines RS3PE-Syndroms gestellt, sollten bislang ausgebliebene altersentsprechende Malignom-Screening-Untersuchungen nachgeholt werden. Zudem sollten eine ausführliche Anamnese und körperliche Untersuchung mit der Frage nach Hinweisen für eine Neoplasie durchgeführt werden, gegebenenfalls gefolgt von weiteren Untersuchungen. Das RS3PE-Syndrom spricht typischerweise exzellent auf eine Therapie mit Glukokortikoiden an. Häufig wird eine medikamentenfreie Remission erreicht, teils ist eine Therapie mit DMARDs („disease-modifying antirheumatic drugs“) notwendig.

## Abstract (en)

The remitting seronegative symmetric synovitis with pitting edema (RS3PE) syndrome is a rare inflammatory rheumatic disease of older adults, which can also be paraneoplastic. The main symptom is a rapidly progressing symmetric edematous swelling of both hands, sometimes also both feet, accompanied by (teno-)synovitis. Autoimmune serologies (rheumatic factor, antibodies against cyclic citrullinated peptides, antinuclear antibodies) typically remain negative. When a diagnosis of RS3PE is made, age-appropriate screening investigations for malignancies should be carried out. In addition, a comprehensive history should be taken and physical examination should be performed focusing on signs of neoplasia, followed by further investigations if necessary. The RS3PE syndrome typically shows an excellent response to treatment with glucocorticoids. A drug-free remission is frequently achieved but sometimes treatment with disease-modifying antirheumatic drugs (DMARDs) is necessary.

## Anamnese

Ein 85-jähriger Patient stellte sich wegen symmetrischer Schwellungen und Schmerzen in beiden Händen und Füßen in der Notaufnahme vor. Die Symptomatik hätte vor 7 Tagen recht plötzlich innerhalb eines Tages begonnen und halte seither an. Ein Trauma war nicht erinnerlich. Die Schmerzen seien nachts besonders ausgeprägt, es folge daraufhin eine Morgensteifigkeit von mehreren Stunden. Die Einnahme von Ibuprofen habe nur eine minimale Besserung der Beschwerden gebracht. Fieber oder sonstige neue Begleitbeschwerden wurden verneint.

Fünf Jahre zuvor war ein High-risk-Prostatakarzinom mit einer Radiohormontherapie behandelt worden, seither sei die Erkrankung anamnestisch in Remission. Es bestand eine Dauerversorgung mit suprapubischem Blasenkatheter bei Strahlentherapie-assoziierten Strikturen des Harntrakts. Bezüglich altersentsprechender Tumorscreeninguntersuchungen war der Patient abgesehen von einer ausstehenden Koloskopie auf dem aktuellen Stand und berichtete über unauffällige Ergebnisse.

An weiteren Vorerkrankungen bestanden zudem: Vorhofflimmern, arterielle Hypertonie, Herzinsuffizienz (New York Heart Association[NYHA]-II), chronisch obstruktive Lungenerkrankung. Folgende Medikamente wurden eingenommen: Enalapril, Metoprolol, Empagliflozin, Torasemid, Apixaban, Amlodipin, Vitamin D, L‑Methionin sowie diverse Inhalativa.

## Klinischer Befund

**Abb. 1:**
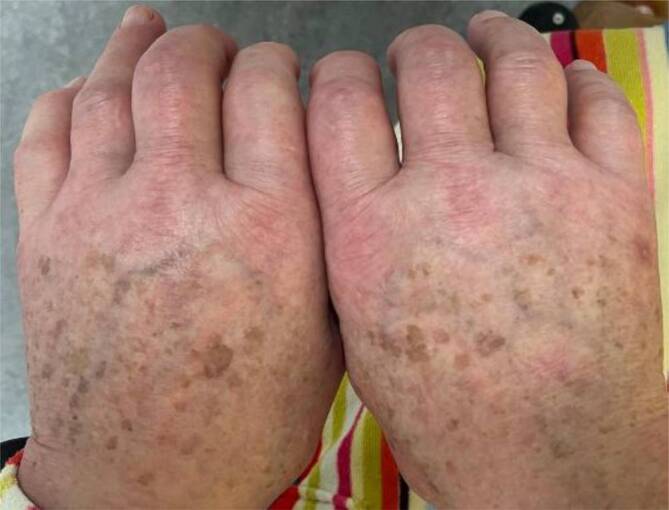
Hände des Patienten. Man sieht eine symmetrische Schwellung beider Handrücken und aller Finger. Die Schwellungen über den Handrücken waren eindrückbar

Der Patient war afebril (36,1 °C), in gutem Allgemeinzustand, wach und orientiert, leicht hypertensiv und tachykard (152/68 mm Hg; 96/min). Im allgemeininternistischen Status gab es keine Auffälligkeiten abgesehen von einem arrhythmischen Puls bei bekanntem Vorhofflimmern. Die Halsvenen waren bds. nicht gestaut. Beide Hände und Füße waren mit eindrückbaren Ödemen diffus geschwollen (Abb. 1), je bis zum Hand- bzw. Sprunggelenk reichend. An beiden Händen und Füßen lag ein ubiquitärer Druckschmerz mit Betonung der Streckseiten von MCP-/MTP- und PIP-Gelenken vor. Das Gaenslen-Zeichen war positiv. Es lagen keine Heberden- oder Bouchard-Knoten vor. Prätibiale Ödeme bestanden nicht.

## Labor

Laborchemisch zeigte sich ein moderat erhöhtes C‑reaktives Protein (14,3 mg/l; Referenzwert < 5,0) bei normwertigem Procalcitonin. Im Differenzialblutbild zeigte sich eine geringgradige Leukopenie (3,59/ml; Referenzwert 3,9–10,5) begleitet von einer leichten Thrombopenie (139/nl; Referenzwert 150–370) bei normwertigem Hämoglobin. Das Thyreoidea-stimulierende Hormon lag im Normbereich. Das N-terminal pro-B-type natriuretic peptide als Marker einer kardialen Druckbelastung sprach bei nur geringer Erhöhung (1109 ng/l; Referenzwert < 697 ng/l) gegen eine höhergradige kardiale Dekompensation. Der Urinstix war unauffällig. Rheumafaktoren (RF), Antikörper gegen zyklische citrullinierte Peptide (ACPA) und antinukleäre Antikörper (ANA) waren allesamt im Normbereich. In der Serumelektrophorese fand sich eine geringe Hypergammaglobulinämie (21,7 %; Referenzwert 11,1–18,8) bei unauffälliger Immunfixation. Das prostataspezifische Antigen lag im unteren Normbereich (0,12 ng/ml; Referenzwert < 4,0).

### Bildgebung

Das Röntgenbild vom Thorax zeigte einzig ein gering global vergrößertes Herz ohne Zeichen einer pulmonalvenösen Stauung und verblieb ohne Nachweis einer suspekten Raumforderung. In einer Sonographie des Abdomens konnte ebenfalls keine relevante Pathologie identifiziert werden. In Röntgenbildern von Händen und Füßen zeigten sich keine Erosionen bei Zeichen der Fingergelenkpolyarthrose.

## Diagnose

RS3PE(„remitting seronegative symmetric synovitis with pitting edema“)-Syndrom.

## Diskussion

Das RS3PE-Syndrom ist eine seltene rheumatische Erkrankung, die paraneoplastisch auftreten kann. Die Diagnose wird klinisch gestellt. Die betroffenen Patienten sind zumeist über 60 Jahre alt, Männer sind häufiger betroffen als Frauen [1].

Leitsymptom ist die zumeist akut beginnende und rasch progrediente, symmetrische ödematöse Schwellung beider Hände, teils auch beider Füße, begleitet durch (Teno-)Synovitiden [1]. Selten (ca. 5 % der Fälle) finden sich auch asymmetrische, einseitige Verläufe [1]. Die (Teno-)Synovitiden finden sich betont an den Streckseiten von Händen und Füßen, Beugeseiten sind geringer betroffen. Mit Sonographie oder Magnetresonanztomographie lassen sich diese Strecksehnentenosynovitiden nachweisen, begleitet von einem subkutanen Ödem und Synovitiden v. a. in den MCP-/MTP- und PIP-Gelenken. Erosionen sind beim RS3PE-Syndrom üblicherweise nicht vorhanden.

Laborchemisch findet sich zumeist eine moderate Entzündungskonstellation mit erhöhtem C‑reaktivem Protein und erhöhter Blutsenkungsgeschwindigkeit, während Autoimmunserologien (RF, ACPA, ANA) typischerweise negativ bleiben, wenn auch einzelne Fälle mit gering erhöhten RF- und ANA-Titern in der Literatur beschrieben werden [2–4]. Rheumatologische und nichtrheumatologische relevante Differenzialdiagnosen des RS3PE-Syndroms sind in Tab. 1 aufgeführt.

**Tab. 1:** Ausgewählte Differenzialdiagnosen des RS3PE-Syndroms

Differenzialdiagnose	Merkmale
(„Late onset“) Seronegative rheumatoide Arthritis	Oft langsamer Beginn
	Ebenfalls negative RF und ACPA sowie Entzündungskonstellation im Labor
	Ggf. Erosionen im konventionellen Röntgenbild
	Typischerweise kein Ödem
Polymyalgia rheumatica	Ebenfalls sehr gutes Ansprechen auf GC sowie Entzündungskonstellation im Labor
	Eher Schulter- und Beckengürtel betroffen
	Typischerweise kein Ödem
Hydropische Dekompensation (z. B. Herzinsuffizienz, akute Nierenschädigung)	Ödem eher nicht beschränkt auf Hände/Füße
	Begleitsymptome, z. B. Dyspnoe bei Lungenödem
	Bei reiner hydropischer Dekompensation ohne begleitenden Infekt keine Entzündungskonstellation im Labor
	Zusätzlich laborchemische Befunde wie beispielsweise Erhöhung von NT-proBNP oder Kreatinin
Myxödem	Ödem nicht beschränkt auf Hände/Füße und nicht eindrückbar
	Laborchemisch ausgeprägte TSH-Veränderung
Chronisch venöse Insuffizienz	Vor allem untere Extremität betroffen
	Keine Entzündungskonstellation im Labor
	Sonographie der Venen zur Differenzierung
Lymphödem	Eher einseitig, insbesondere bei sekundärem Auftreten nach z. B. Lymphknotenexstirpation
	Keine Entzündungskonstellation im Labor
	Klinisch ggf. positives Stemmer-Zeichen
Kristallarthropathien (Gicht, Chondrokalzinose)	Eher einseitig, häufig mit Rötung und starken Schmerzen
	Nachweis von Kristallen in der Synovialflüssigkeit

*RF* Rheumafaktor, *ACPA* Antikörper gegen zyklische citrullinierte Peptide, *GC* Glukokortikoide, *NT-proBNP* N-terminal pro-B-type natriuretic peptide, *TSH* Thyreoidea-stimulierendes Hormon

Beim RS3PE-Syndrom fanden sich in systematischen Literaturrecherchen begleitende Neoplasien in ca. 16–20 % aller Fälle [1, 5]. Diese Neoplasien können dem RS3PE sowohl vorausgehen als auch nachfolgen. Wird die Diagnose eines RS3PE-Syndroms gestellt, so sollten bislang ausgebliebene altersentsprechende Tumorscreeninguntersuchungen nachgeholt werden. Zudem sollten eine ausführliche Anamnese und körperliche Untersuchung mit der Frage nach Hinweisen für eine Neoplasie durchgeführt werden, ggf. gefolgt von weiteren Untersuchungen.

## Therapie und Verlauf

Das RS3PE-Syndrom spricht typischerweise exzellent auf eine Therapie mit Glukokortikoiden (GC) an. In der Literatur werden zumeist Startdosierungen von 15–20 mg/Tag Prednisolon genannt. Häufig wird hiermit im Verlauf von einigen Monaten eine medikamentenfreie Remission erreicht, teils ist eine Therapie mit DMARDs („disease-modifying antirheumatic drugs“) notwendig. In Fallberichten wurden hier beispielsweise Methotrexat [6, 7] oder Hydroxychloroquin [5, 8] angewandt. Ein schlechtes Ansprechen auf GC kann ein Hinweis auf eine Neoplasie oder eine Fehldiagnose sein, ebenso das Wiederauftreten des RS3PE-Syndroms im Verlauf [1]. In einer Nachbeobachtung von 22 Patienten mit RS3PE-Syndrom in Spanien waren nach 6 Jahren 13 Patienten in medikamentenfreier Remission, während 9 (41 %) in der Zwischenzeit eine Therapie mit DMARDs beginnen mussten [9]. Bei 4 der 22 Patienten (18 %) wurde im Verlauf eine andere rheumatologische Diagnose (2 Patienten mit seronegativer rheumatoider Arthritis; Polymyalgia rheumatica und systemische Sklerose je ein Patient) gestellt.

Die Beschwerden unseres Patienten waren nach einigen Tagen oraler Prednisolon-Einnahme (20 mg/Tag) bereits deutlich gebessert, sodass wir ihn mit ambulanter Anbindung und einem GC-Dosisreduktionsschema sowie einer Empfehlung zur ambulanten Koloskopie entlassen konnten.

## Take-Home-Message

Beim älteren Patienten mit akut beginnender, symmetrischer, schmerzhafter, ödematöser Schwellung beider Hände und ggf. Füße sollte an das RS3PE-Syndrom gedacht werden. Die Entzündungsparameter sind erhöht, während Autoimmunserologien typischerweise unauffällig bleiben. Wird die Diagnose gestellt, so sollte man gezielt nach Neoplasien fahnden. Das RS3PE-Syndrom spricht üblicherweise sehr gut auf GC an.
